# Supplementary material for: NAD(P) transhydrogenase has vital non‐mitochondrial functions in malaria parasite transmission
Source: EMBO Rep. 2020 Jan 17;21(3):e47832. doi: 10.15252/embr.201947832 (PMC7054674; doi:10.15252/embr.201947832)
Supplement: Supplementary file 1 — Expanded View Figures PDF [file EMBR-21-e47832-s001.pdf]

Expanded View Figures

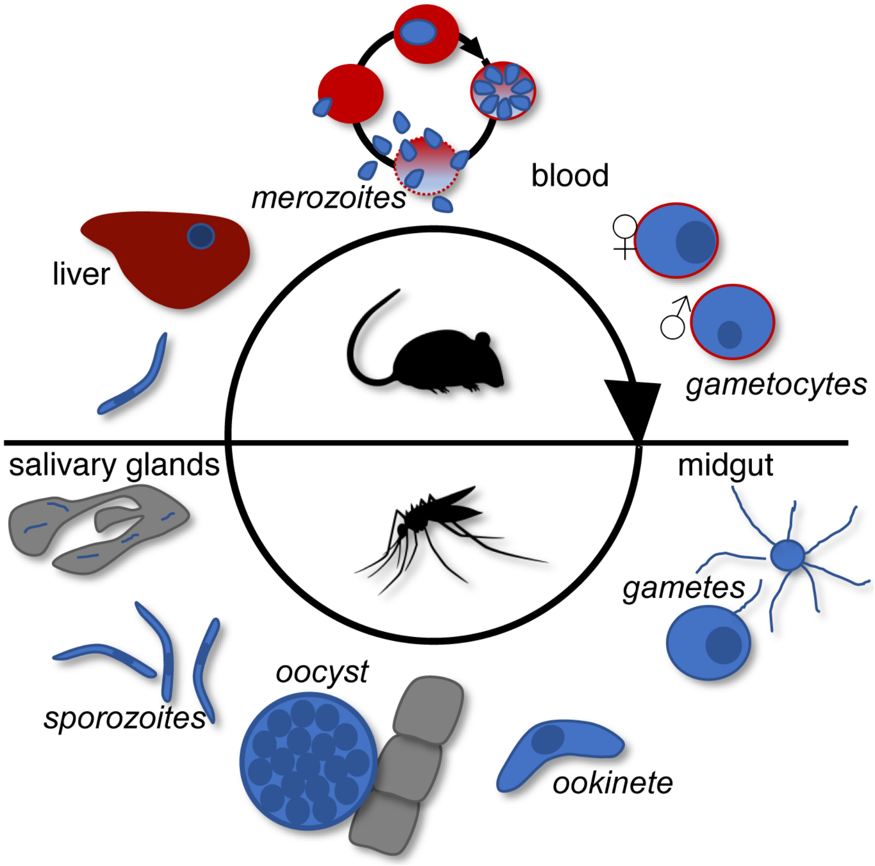

Figure EV1. Life cycle of malaria parasites in mouse and mosquito.

Parasites are depicted blue, mosquito tissues grey, liver dark red, and red blood cells red.

 © 2020 The Authors

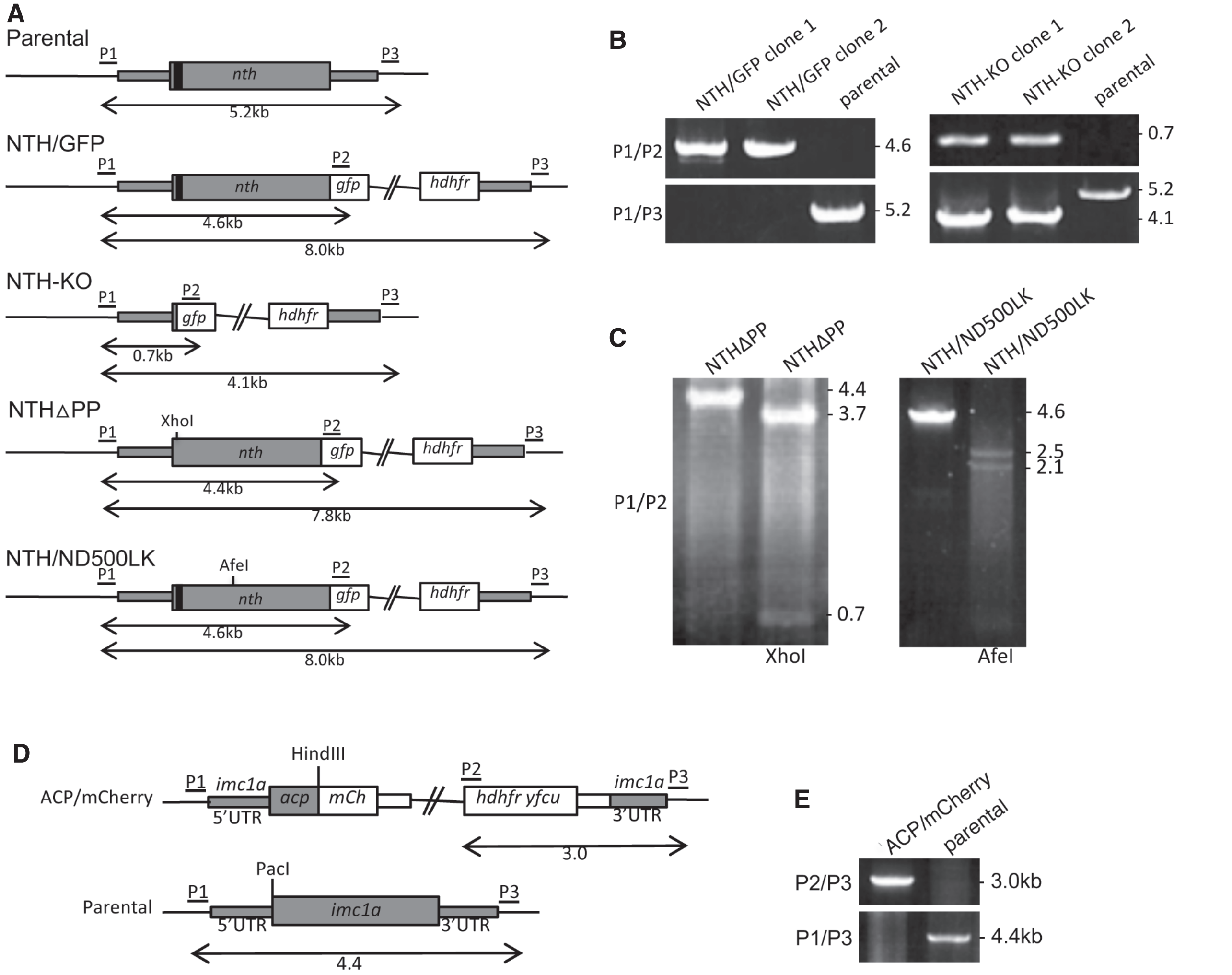

Figure EV2. Generation of genetically modified parasite lines.

A Schematic diagram of the unmodified (parental) and modified *nth* alleles in parasite lines NTH/GFP, NTH-KO, NTHΔPP, and NTH/ND500LK. The *nth* gene is indicated with coding sequence (wide grey bars, introns not shown) and 5′ and 3′ untranslated regions (UTRs; narrow grey bars). Also indicated are the relative positions of the putative apicoplast targeting signal (black box), GFP module (*gfp*); the human DHFH selectable marker gene cassette (*hdhfr*); the XhoI and AfeI diagnostic restriction sites; and primers used for diagnostic PCR amplification (P1–P4). Sizes of PCR products are also indicated.

B Diagnostic PCR of parasite lines NTH/GFP and NTH-KO. Integration of the modified GFP-tagged alleles into the target locus across the 5′ integration site was carried out with primers P1 (ATATTTCCCCCTAATTTTCCCTT) and P2 (GTGCCCATTAACATCACC), amplifying expected products of ~ 4.6 kb in NTH/GFP and 0.7 kb in NTH-KO. Absence of the unmodified *nth* allele was carried out with primers P1 (ATATTTCCCCCTAATTTTCCCTT) and P3 (ATTTCAATACTCGAATTTATGTTATCG), amplifying expected products of ~ 5.2 kb from the parental parasite and 4.1 kb in NTH-KO parasites.

C Diagnostic PCR and restriction enzyme digests of parasite lines NTHΔPP and NTH/ND500LK. Primers P1 and P2 amplify a 4.4 kb product in NTHΔPP that is digested by XhoI into the expected two fragments of ~ 3.7 kb and 0.7 kb, confirming presence of the mutation. Primers P1 and P2 amplify a 4.6 kb product in NTH/ND500LK that is digested by AfeI into the expected two fragments of ~ 2.5 kb and 2.1 kb, confirming presence of the mutation.

D Schematic diagram of the modified *acp* allele (PBANKA_0305600) in parasite line ACP/mCherry. The *acp* gene is indicated with coding sequence (wide grey bars, introns not shown) and *imc1a* 5′ and 3′ untranslated regions (UTRs; narrow grey bars). Also indicated are the relative positions of the mCherry module (*mCh*); the human DHFR::yFCU selectable marker gene cassette (*hdhfr yfcu*); and primers used for diagnostic PCR amplification (P1–P3). Sizes of PCR products are also indicated.

E Diagnostic PCR for integration into the target *imc1a* locus with primers P2 and P3, giving rise to a 3.0 kb product (top panel). Diagnostic PCR with primer pair P1 and P3 amplified an ~ 4.4 kb fragment from the parental (wild-type) parasites, and no product in the ACP/mCherry parasites, confirming absence of the unmodified *imc1a* allele in the clonal transgenic parasite line (bottom panel).

© 2020 The Authors

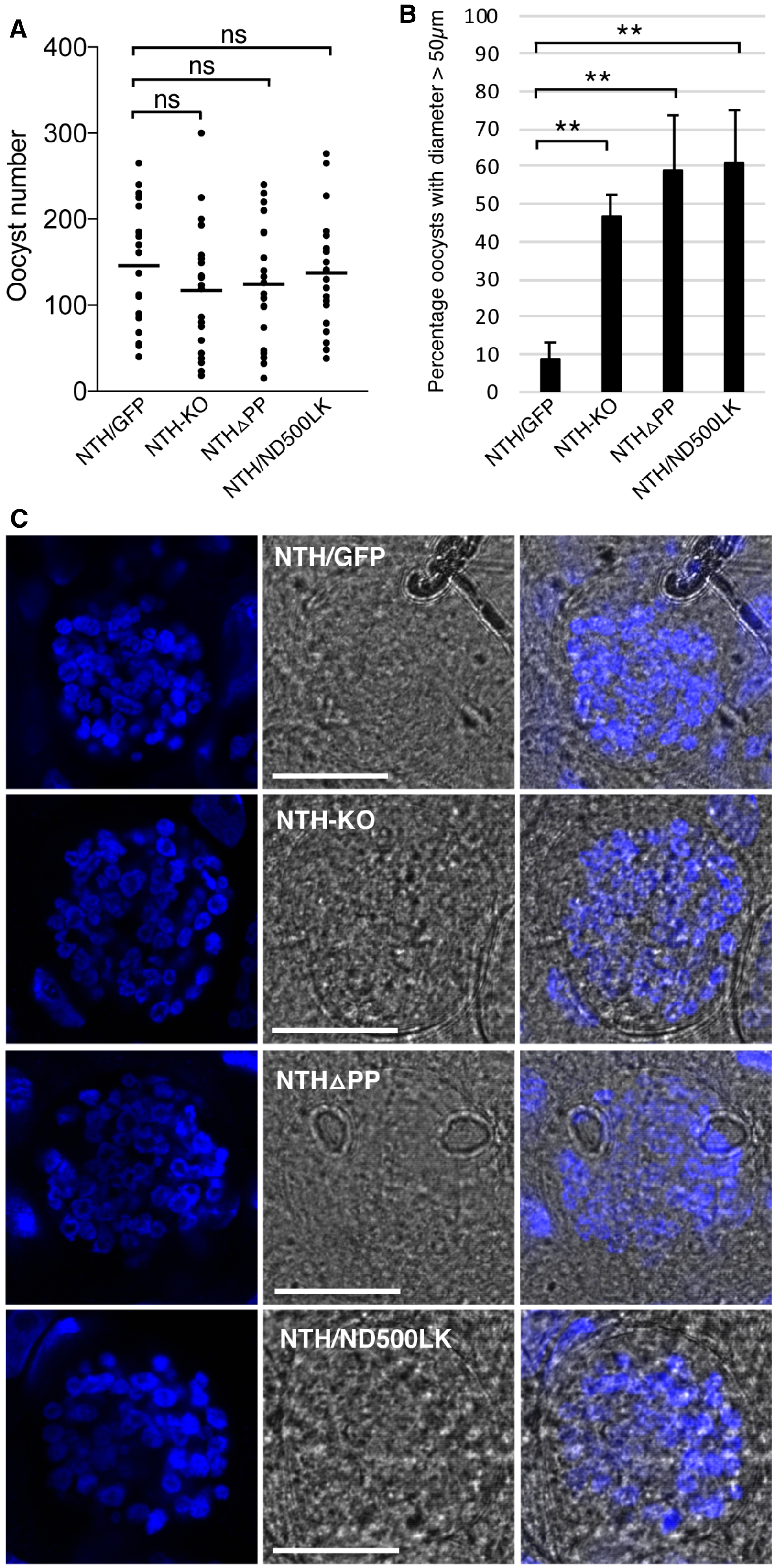

Figure EV3. Oocyst development in *Anopheles stephensi* infected with parasite lines NTH/GFP, NTH-KO, NTH∆PP, and NTH/ND500LK.

A Aligned dot plot of oocyst numbers at 10 days post-infection. Horizontal lines mark mean values (n = 20); ns, not significantly different (Mann–Whitney).

B Bar chart showing percentage of oocyst with a diameter > 50 µm at 15 days post-infection. Diameters were scored at 400× magnification using an eyepiece micrometre. Five infected mosquitoes and 100 oocysts per mosquito were sampled for each parasite line. Error bars show standard deviations (n = 5). ** mark statistically significant differences (P < 0.001, Mann–Whitney); ns, not significant.

C Brightfield and fluorescence images of oocysts pre-sporulation 11 days post-infection, stained with Hoechst DNA stain (blue), showing similar developmental progression between NTH/GFP and mutants. Scale bar = 20 µm.

 © 2020 The Authors

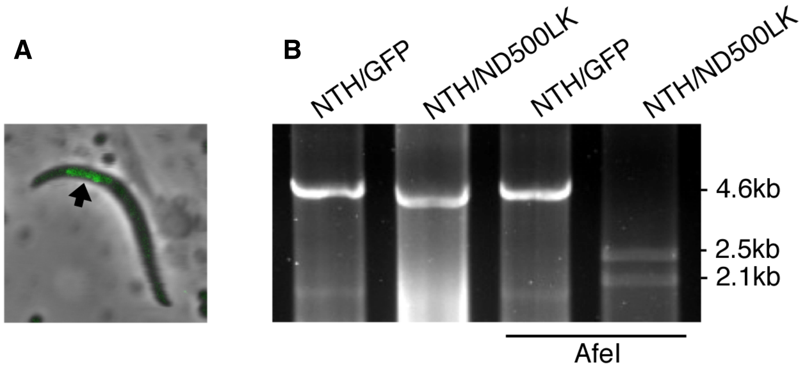

Figure EV4. NTH/ND500LK sporozoites can initiate intraerythrocytic infection in mice.

A Merged brightfield and fluorescence image of a sporozoite of parasite line NTH/ND500LK showing localisation of NTH::GFP in the apicoplast (arrow).

B PCR diagnostic for the presence of modified *nth* alleles in blood-stage infections obtained after sporozoite transmission of NTH/ND500LK or NTH/GFP sporozoites, amplifying an ~ 4.6 kb fragment. The NTH/ND500LK fragment contains an AfeI restriction site, diagnostic of the point mutation introduced into its *nth* allele (see also Fig EV2).

© 2020 The Authors
